# Cooperative Detection of Multiple Targets by the Group of Mobile Agents

**DOI:** 10.3390/e22050512

**Published:** 2020-04-30

**Authors:** Barouch Matzliach, Irad Ben-Gal, Evgeny Kagan

**Affiliations:** 1 Department Industrial Engineering, Tel-Aviv University, Tel-Aviv 6997801, Israel; bengal@tauex.tau.ac.il; 2 LAMBDA Laboratory, Tel-Aviv University, Ramat Aviv 6997801, Israel; evganyk@ariel.ac.il; 3 Department Industrial Engineering, Ariel University, Ariel 40700, Israel

**Keywords:** search and detection, multi-agent systems, probabilistic decision-making, information gain, stochastic learning, probabilistic search

## Abstract

The paper considers the detection of multiple targets by a group of mobile robots that perform under uncertainty. The agents are equipped with sensors with positive and non-negligible probabilities of detecting the targets at different distances. The goal is to define the trajectories of the agents that can lead to the detection of the targets in minimal time. The suggested solution follows the classical Koopman’s approach applied to an occupancy grid, while the decision-making and control schemes are conducted based on information-theoretic criteria. Sensor fusion in each agent and over the agents is implemented using a general Bayesian scheme. The presented procedures follow the expected information gain approach utilizing the “center of view” and the “center of gravity” algorithms. These methods are compared with a simulated learning method. The activity of the procedures is analyzed using numerical simulations.

## 1. Introduction

Methods of search and detection address various problems of finding hidden objects and chasing after targets [1]. Studies in this field were initiated in 1942 as a part of the mission to detect submarines in the Atlantic [2] and were later distributed among various applications and scenarios.

In particular, the search problem addresses the activity of the searcher up to hunting the target in its location, and often results in an optimal search policy or in an effective movement trajectory of the searcher. The detection problem, in contrast, focuses on the recognition of the target’s location without necessarily reaching its physical location and results, usually, in a cost-effective distribution of the search efforts [2]. For an overview of the field and related problems, see, e.g., [3,4,5]. In the last decades, with the development of mobile robots and multi-robot systems, methods of search and detection were extended to apply to groups of autonomous agents, so the current studies also include considerations of communication and collective decision-making under uncertainties [6,7].

In the paper, we consider the problem of detection of multiple targets by a group of mobile agents. This problem is a direct extension of the classical Koopman setting that aims at the detection of the hidden objects [2,5,8]. However, in contrast to Koopman’s formulation, we assume that the detection process is conducted by a finite small number of indivisible agents that start in certain locations, move over the domain, and explore it up to the detection of all the targets. Also, we assume that the agents are equipped with sensors that can detect the existence of the target in certain locations, yet with both false positive and false negative errors. The simple version of this problem was considered in 2012 by Israel et al. [9] in the framework of search in shadowed space. In the same year, Chernikhovsky et al. [10] considered a similar search problem with erroneous sensors and showed that the solution to the detection algorithm terminates in a finite number of steps.

The search problem after static or moving targets by finite and usually small number of agents appears in various applications, both military, and civil ones. The standard taxonomies of this problem are often based on the targets’ and the search agents’ abilities, such as their mobility level, their knowledge about the activities of the other party and on their cooperation level [4,7,11]. Many search algorithms are classified with respect to the optimization principles that govern the motion of the agents in the group. In particular, since a global optimization of the agents’ motions requires unreasonable time and computation power, search algorithms are often implemented by using different heuristics, mostly informational one or by mimicking animal foraging [6,7,12].

In the paper, we consider a detection process with several assumptions, which are usually considered separately. Following the basic Koopman formulation, we consider a probabilistic search scheme, in which the search agents have knowledge only about the targets’ location probabilities, while assuming that Koopman’s exponential random search formula which defines those detection probabilities is applicable. At the same time, we assume that the search-agents’ group includes a finite small number of members and that the agents are indivisible; such an assumption implies that the problem cannot be considered solely as a search-efforts distribution problem, but also as a problem that requires methods of swarm navigation and control.

In practice, communication among the agents as well as information processing can be organized at different levels: from peer-to-peer networks to a scheme of a central station that receives information from all the agents in the group [6,7]. In the first case, each agent obtains information from its neighbors and makes decisions based on such local information, while in the second case, a central station defines the agents’ motion based on global information. In practice, the algorithms of swarm control use both approaches, while in practice, applications based on a central station are usually restricted by computation power and communication constraints. Also, in most of the military applications, the use of a central station is a challenging one due to security reasons. In the suggested techniques, we assume an existence of a central station that holds a global probability map, which, on the one hand, can be used for theoretical consideration of the effectiveness of the suggested methods, while, on the other hand, is required for generating strict criteria for the termination of the detection process. Nonetheless, as we demonstrate in the paper, the use of a global map by a central station for the navigation of the group of search agents is less effective than the use of local maps accompanied by peer-to-peer communication.

Finally, we continue a line of practical research works [10,13], yet in contrast to some known methods of group search and detection, we consider a more realistic situation, in which both false positive and false negative detection errors exist. Another practical assumption is the one that considers a variety of sensors that can be used by each agent.

In this study, we consider the detection of a number of static targets; however, the developed algorithms allow further modification for the detection of mobile targets that is out of the scope of this paper. The objective of the presented research is to introduce methods of control of the mobile agents acting within a group such that detection of the targets is conducted in a minimal time period. Notice that the agents are not required to catch the targets, which is to reach physically their locations, but rather to detect the locations of the targets using their on-board sensors.

The suggested solution follows the occupancy grid approach, where the map of the targets’ candidate points is created simultaneously with the detection process and the agents’ motion [14,15]. The implemented sensor-fusion scheme follows a general Bayesian scheme [16] with varying sensitivity of the sensors.

The algorithm implements three different levels of the agent’s knowledge about the targets’ location:

- A global map that represents the information that is available to the group of agents and is obtained by fusion of information which is available to each agent.
- A local map that represents the information available to every single agent and is obtained by fusion of information obtained by the agent’s sensors.
- A sensor map which is obtained by a single sensor.

The above maps are also called probability maps since they provide the information on the target’s location by a probability distribution, often using a colored heat map to indicate the probability of the target being located in each grid of the map.

The algorithm was trained with different decision-making objectives based on:

- The expected information gain by the agent’s next step.
- The location of the center of view, which indicates a future agent’s location that, given the sensors’ capabilities, is expected to yield a maximal modification of the probability map. Formally, this approach relies on the expected information gain procedure which is applied to the global map instead of focusing on the close neighborhood of the agent.
- The location of the center of gravity of the map, which is the first moment of the targets’ location probabilities.

In the detection by a single agent, it was found that all three procedures provide similar results. However, since the center of view approach implements additional information about sensors’ capabilities, in certain cases, it demonstrates better performance than two other algorithms. Also notice, that if the sensors are errorless and equal, then the center of view and the center of gravity approaches result in the same detection times.

In a collective detection by the agents’ group, it was found that in all three algorithms, the use of an individual local map by each agent results in shorter detection times than the times when using the global map. Further studies of these scenarios demonstrated that, due to the similar decisions which are governed by the use of a global map, the agents move towards the same areas instead of dividing the efforts over the space to simultaneously investigate different areas. These results meet recent theoretical considerations of the altruistic and egoistic behavior of search agents in the groups [17] and form a basis for further considerations of the problem of “division of labor” in the groups of autonomous agents. 

The algorithm was implemented by Python programming language, and the code can be directly used for solving the real-world tasks of detection of targets by groups of mobile agents.

## 2. Scenarios of Cooperative Detection

The considered detection problem follows general Koopman’s scenario [2] (see also [5,8]) with additional consideration of the agents’ motion toward the destinated locations. Formally, using the occupancy grid approach [14,15], the problem is defined as follows.

Let $C=\left\{c_{1},c_{2},\ldots ,c_{n}\right\}$ be a finite set of cells such that C represents a grid over a closed two-dimensional domain, and consider a set of 1≤m≪n mobile agents $A_{j}$, j=1, 2, …, m, searching for hidden targets in the domain. For simplicity, we assume that each agent, as well as each target, can occupy only a single cell of the grid.

The state of a cell $c_{i}$, i=1, 2, …,n is defined as a discrete random variable taking values $s_{i}=s\left(c_{i}\right)\in \left\{0,1\right\}$, such that $s_{i}=0$ implies that the cell $c_{i}$ does not contain any target, while $s_{i}=1$ implies that cell $c_{i}$ contains a target. In case we need to stress the time *t* of the sensing, we will use the notation $s_{i}^{t}=s\left(c_{i},t\right)$, otherwise, we omit it. Note that, at any time t and for each cell $c_{i}$, the probabilities of these events are mutually exclusive, i.e.,

(1) $$Pr\left\{s_{i}=0\right\}+Pr\left\{s_{i}=1\right\}=1,$$

Each agent $A_{j}$ is equipped with a variety of sensors ${𝕤}_{jk}$, k=1,2,…l, that provide, not necessarily accurate, information about the states of the cells $s_{i}$, i=1, 2, …,n, relative to the agent’s distance with respect to the Koopman’s exponential random search Formula [2],

(2) $$Pr\left\{target\;detected\;in\;c_{i}\;|\;target\;located\;in\;c_{i}\right\}=\exp\left[-\theta \left(d\left(c_{i},c_{j}\right),\tau \right)\right],$$

where $\theta \left(d\left(c_{i},c_{j}\right),\tau \right)$ represents the search effort applied to the cell $c_{i}$ with respect to the distance $d\left(c_{i},c_{j}\right)$ between this cell $c_{i}$ and the agent’s location $c_{j}$ for observation period τ. It is assumed that as the distance $d\left(c_{i},c_{j}\right)$ gets shorter and as the period τ gets longer, the higher the detection probability will be.

In order to formalize the possibility of both false positive and false negative detection errors, let us assume that the domain includes both true and dummy targets that broadcast signals indicating their presence in the domain cells. The signals sent by the true targets are considered as true alarms, and the signals that are sent by the dummy targets are considered as false alarms that represent the false-positive errors.

Then, with respect to Koopman’s search Formula (2), the probability of a perceived alarm is defined as follows:

(3) $$Pr\left\{alarm\;percieved\;|\;alarm\;sent\right\}=\exp\left[-d\left(c_{i},c_{j}\right)/\lambda_{jk}\right],$$

where $\lambda_{jk}=\lambda \left({𝕤}_{jk}\right)$ is the sensitivity of the sensor ${𝕤}_{jk}$ installed on agent $A_{j}$. The dependence of the detection probability at observation period τ is considered in the updates of the probability map as defined below.

The probabilities $p_{i}=p\left(c_{i}\right)=Pr\left\{alarm\;sent\;from\;c_{i}\right\}$ of sending alarms from cells $c_{i}\in C$, i=1,2,…,n, are defined by the probability map that represents the information about the targets’ locations in the domain. Moreover, we assume that the agents can share information about the targets’ locations as they have been perceived by the sensors.

The activity of the agents is outlined as follows. The agents start with some initial probability map

$$P\left(t\right)=\left\{p_{1}\left(t\right),p_{2}\left(t\right),\ldots ,p_{n}\left(t\right)\right\},$$

that defines initial probabilities $p_{i}\left(t\right)$ of detecting the targets in cells $c_{i}\in C$, i=1,2,…,n, at time t.

At time t, the agents $A_{j}$, j=1, 2, …, m, are located in the cells $c_{j}\left(t\right)$ and obtain the sent signals (that are either true or false alarms) from the cells in which the targets can be located. The probabilities of receiving the signal that was sent from the cell $c_{i}$ with probability $p_{i}$ is defined by the Koopman Formula (3).

After receiving the signals, the jth agent $A_{j}$ updates the sensor probability maps, k=1, 2, …, l,

$$P^{sensor}\left(j,k,t\right)=\left\{p_{s_{1}=1}^{sensor}\left(j,k,t\right),\;p_{s_{2}=1}^{sensor}\left(j,k,t\right),\ldots ,p_{s_{n}=1}^{sensor}\left(j,k,t\right)\right\},$$

where

$$p_{s_{i}=1}^{sensor}\left(j,k,t\right)=Pr\left\{sensor\;{𝕤}_{jk}\;identifies\;a\;true\;target\;in\;c_{i}\;at\;time\;t\right\}.$$

The resulting sensor probability maps $P^{sensor}\left(j,k,t\right)$, k=1, 2, …, l, are combined into the probability map

$$P^{agent}\left(j,t\right)=\left\{p_{s_{1}=1}^{agent}\left(j,t\right),\;p_{s_{2}=1}^{agent}\left(j,t\right),\ldots ,p_{s_{n}=1}^{agent}\left(j,t\right)\right\},$$

where probabilities $p_{s_{i}=1}^{agent}\left(j,t\right)$ of the target’s locations in the cells $c_{i}$, i=1, 2, …,n, from the agent’s point of view are specified by fusion of the sensors’ probability maps $P^{sensor}\left(j,k,t\right)$, k=1, 2, …, l. 

Finally, a global probability map

$$P^{global}\left(t\right)=\left\{p_{s_{1}=1}^{global}\left(t\right),\;p_{s_{2}=1}^{global}\left(t\right),\ldots ,p_{s_{n}=1}^{global}\left(t\right)\right\},$$

that defines the probabilities $p_{s_{i}=1}^{global}\left(t\right)$ of the target’s locations in the cells $c_{i}$, i=1, 2, …,n, as they are known by the group of agents is obtained by the fusion of the agents’ probability maps $P^{agent}\left(j,t\right)$ over all the agents $A_{j}$, j=1,2,…,m.

In the presented algorithms, the probability maps both at the sensors’ level and at the agents’ level are fused using a simple Bayesian scheme. Exact equations for calculating each probability map are presented in the next sections.

The general scenario of the targets’ detection by a group of mobile agents is outlined as follows. At each step, each agent makes a decision regarding its own next movement. The agent’s decision is based on either the local or the global probability maps (or both of them) as obtained at this step.

After taking its decision, the agent makes a movement step towards the chosen direction. At the completion of the step and arrival at the required cell, the agent observes the cells of the domain by utilizing its sensors, obtains true or false information about the target’s location, and updates the probability maps, respectively.

Then, the detection process continues following the updated probability maps and the agent’s current locations, in a step forward manner.

Our goal is to define the trajectories of the agents over the domain, such that all the targets will be detected in minimal time. Notice again that we do not require the agents to arrive physically to the exact targets’ locations, but rather to detect the locations of the targets at some level of certainty.

It is clear that the formulated problem follows the general Koopman’s scenario [2]. As defined in the framework of probabilistic search [5,16]. Since the general case, the computational complexity of finding the optimal solution is $O\left(n^{m}\right)$, we are interested in a practically computable near-optimal solution. In the next section, we consider several heuristic approaches and reasonable assumptions that lead to such a solution.

## 3. Sensor Fusion and Updating Schemes over the Probability Maps

As indicated above, we assume that each agent $A_{j}$ is equipped with several sensors ${𝕤}_{jk}$, k=1,2,…l, that independently provide, not necessarily accurate, information about the cells states $s_{i}$, i=1, 2, …,n. In the used framework of the occupancy grid, sensor fusion is conducted as follows.

Let for example ${𝕤}_{j1}$ and ${𝕤}_{j2}$ be two independent sensors installed on agent $A_{j}$ and let ${\tilde{s}}_{j1}\left(c_{i},t\right)$ and ${\tilde{s}}_{j2}\left(c_{i},t\right)$ be the signals obtained by these sensors at time t. Then, the probability that the target is located in the cell $c_{i}$, that is the state $s_{i}\left(t\right)=1$, is defined by Bayes rule as follows (see also [15]):

(4) $$Pr\left\{s_{i}\left(t\right)=1\;|\;{\tilde{s}}_{j1}\left(c_{i},t\right)=1,\;{\tilde{s}}_{j2}\left(c_{i},t\right)=1\right\}=\frac{Pr\left\{{\tilde{s}}_{j2}\left(c_{i},t\right)=1│s_{i}\left(t\right)=1\right\}\times Pr\left\{s_{i}\left(t\right)=1|\;{\tilde{s}}_{j1}\left(c_{i},t\right)=1\right\}}{\sum_{s_{i}\left(t\right)}Pr\left\{{\tilde{s}}_{j2}\left(c_{i},t\right)=1│s_{i}\left(t\right)\right\}\times Pr\left\{s_{i}\left(t\right)|\;{\tilde{s}}_{j1}\left(c_{i},t\right)=1\right\}},$$

where the sum is taken over all possible values of $s_{i}\left(t\right)$. In the considered case, these values are $s_{i}\left(t\right)\in \left\{0,\;1\right\}$.

An extension of this equation to l onboard sensors of the agent $A_{j}$ results in the probabilities

(5) $$p_{s_{i}=1}^{agent}\left(j,t\right)=\frac{\prod_{k=1}^{l}p_{s_{i}=1}^{sensor}\left(j,k,t\right)}{\prod_{k=1}^{l}p_{s_{i}=1}^{sensor}\left(j,k,t\right)+\prod_{k=1}^{l}\left(1-p_{s_{i}=1}^{sensor}\left(j,k,t\right)\right)},$$

of the targets’ locations in the cells $c_{i}$, i=1, 2, …,n, as they determined by the agent $A_{j}$ using its sensors. This equation is based on the approach known as “independent opinion pool” [15] under the assumption that the sensors are conditionally independent and that their reliabilities and accuracies are equivalent.

Similarly, the location probabilities of different agents can be fused to global probabilities,

(6) $$p_{s_{i}=1}^{global}\left(t\right)=\frac{\prod_{j=1}^{m}p_{s_{i}=1}^{agent}\left(j,t\right)}{\prod_{j=1}^{m}p_{s_{i}=1}^{agent}\left(j,t\right)+\prod_{j=1}^{m}\left(1-p_{s_{i}=1}^{agent}\left(j,t\right)\right)},$$

of the targets’ locations in cells $c_{i}$, i=1, 2, …,n, as determined by the group of the agents. Notice that such a definition requires a central unit that receives data from each agent and computes a global probability map using the obtained probabilities from all the agents.

The presented equations use the probabilities $p_{s_{i}=1}^{sensor}\left(j,k,t\right)$ of the targets’ locations in the cells $c_{i}$, i=1, 2, …,n, as determined by sensors ${𝕤}_{jk}$, k=1,2,…l, installed on agents $A_{j}$, j=1,2,…,m. These probabilities form a sensor probability maps $P^{sensor}\left(j,k,t\right)$ that are updated as follows.

At the initial time t=0 the probabilities $p_{s_{i}=1}^{sensor}\left(j,k,t\right)$ are specified with respect to some initial distribution; if no information is available, these probabilities can be drawn by a uniform distribution. Then, these probabilities are updated by using the Bayesian approach as follows.

As indicated above, let ${\tilde{s}}_{jk}\left(c_{i},t\right)$ be the signal obtained about cell $c_{i}$ relying on sensor ${𝕤}_{jk}$ of agent $A_{j}$ at time t. Recall that in the considered scenario, ${\tilde{s}}_{jk}\left(c_{i},t\right)=1$ implies that cell $c_{i}$ is occupied by a target while ${\tilde{s}}_{jk}\left(c_{i},t\right)=0$ implies that cell $c_{i}$ is empty, both based on sensor ${𝕤}_{jk}$.

Then, the state probabilities of cell $c_{i}$ that are updated by the sensor outputs are:

- if a signal is perceived that is ${\tilde{s}}_{jk}\left(c_{i},t\right)=1$, considering that the static target was located at the cell at time t−1, then the true positive probability is
  (7) $$p_{s_{i}=1}^{sensor}\left(j,k,t\right)=Pr\left\{s_{i}\left(t\right)=1\;|{\tilde{s}}_{jk}\left(c_{i},t\right)=1\right\}=\frac{Pr\left\{s_{i}\left(t-1\right)=1\right\}\times Pr\left\{{\tilde{s}}_{jk}\left(c_{i},t\right)=1│s_{i}\left(t\right)=1\right\}}{\sum_{s_{i}\left(t\right)}Pr\left\{s_{i}\left(t-1\right)\right\}\times Pr\left\{{\tilde{s}}_{jk}\left(c_{i},t\right)=1│s_{i}\left(t\right)\right\}},$$
- Otherwise, while ${\tilde{s}}_{jk}\left(c_{i},t\right)=0$, the false positive probability is
  (8) $$p_{s_{i}=1}^{sensor}\left(j,k,t\right)=Pr\left\{s_{i}\left(t\right)=1\;|{\tilde{s}}_{jk}\left(c_{i},t\right)=0\right\}=\frac{Pr\left\{s_{i}\left(t-1\right)=1\right\}\times Pr\left\{{\tilde{s}}_{jk}\left(c_{i},t\right)=0│s_{i}\left(t\right)=1\right\}}{\sum_{s_{i}\left(t\right)}Pr\left\{s_{i}\left(t-1\right)\right\}\times Pr\left\{{\tilde{s}}_{jk}\left(c_{i},t\right)=0│s_{i}\left(t\right)\right\}}.$$

These equations define an updating scheme of the probabilities map for the sensor given the new observations. They include the probabilities $Pr\left\{{\tilde{s}}_{jk}\left(c_{i},t\right)=1│s_{i}\left(t\right)=1\right\}$ that the sensor perceives the signal given that the target is in the cell $c_{i}$ and the probability $Pr\left\{{\tilde{s}}_{jk}\left(c_{i},t\right)=0│s_{i}\left(t\right)=1\right\}$ that the sensor does not perceive a signal from cell $c_{i}$ given that the target is in that cell.

In order to define these probabilities, denote by $\tilde{a}\left(c_{i},t\right)$ an alarm signal that is sent about cell $c_{i}$ at time t. The value $\tilde{a}\left(c_{i},t\right)=1$ implies, truly or not, that the cell is occupied and the value $\tilde{a}\left(c_{i},t\right)=0$ implies, truly or not, that the cell is empty. Then, implementing Koopman’s Formula (3), one obtains the following

(9) $$Pr\left\{{\tilde{s}}_{jk}\left(c_{i},t\right)=1│s_{i}\left(t\right)=1\right\}=Pr\left\{\tilde{a}\left(c_{i},t\right)=1│s_{i}\left(t\right)=1\right\}\times \exp\left[-d\left(c_{i},c_{j}\right)/\lambda_{jk}\right],$$

(10) $$Pr\left\{{\tilde{s}}_{jk}\left(c_{i},t\right)=1│s_{i}\left(t\right)=0\right\}=1-Pr\left\{{\tilde{s}}_{jk}\left(c_{i},t\right)=1│s_{i}\left(t\right)=1\right\},$$

where, as above, $d\left(c_{i},c_{j}\right)$ is the distance between the cell $c_{i}$ and the agent’s location $c_{j}$ and $\lambda_{jk}=\lambda \left({𝕤}_{jk}\right)$ is the sensitivity of the sensor ${𝕤}_{jk}$ installed on the agent $A_{j}$.

These equations enable to calculate the occupation probabilities at each time t, given the probabilities at the previous time t−1 and the information obtained by the sensors at time t. As indicated above, at the initial time t=0, the probabilities are defined based on topographic data and prior information or, in the worst case, can be specified by a uniform distribution of the occupancy grid.

**Figure 1:**
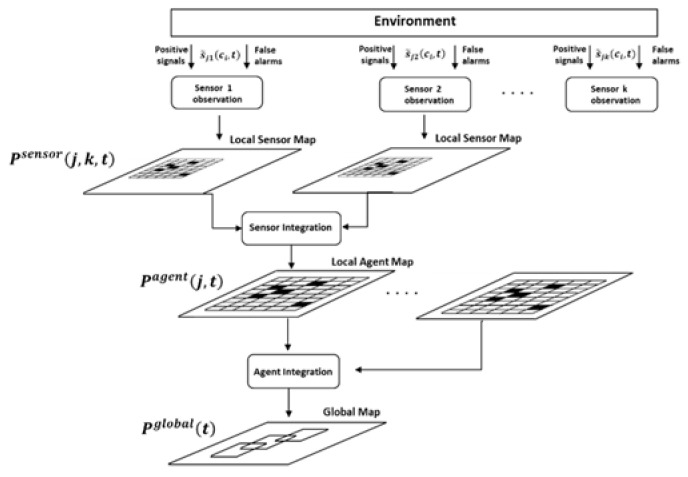
The process of sensors’ fusion.

The above defined process of sensors’ fusion is illustrated in Figure 1.

The sensors receive signals ${\tilde{s}}_{jk}\left(c_{i},t\right)$ from the environment. Part of these signals are positive signals from the targets, indicating the real locations of the targets, while others are false alarms (i.e., false positive errors) that corresponds to false locations of the targets. Based on the received signals for each sensor, a local sensor map is created (see Equations (7) and (8)). Then, each agent integrates its sensor maps to a local agent map (see Equation (5)). Finally, a global map is created by integrating the agent maps (see Equation (6)). Such a hierarchical structure allows us to consider the maps of each level separately and, consequently, to define a more effective calculation process that uses only the maps required for current computations.

## 4. Agents’ Policies and Decision Making

In this section, we define the behavior of the group of agents taking actions in a gridded domain aiming to detect hidden targets. The agents detect the targets by their on-board sensors such that the sensors can identify the targets from certain nonzero distances. The goal is to define the trajectories of the agents such that they detect the targets in minimal time. 

Formally, this problem is defined as follows. Denote by $\tau_{j}\left(T\right)=\left(c_{j}\left(0\right),c_{j}\left(1\right),\ldots ,c_{j}\left(T\right)\right)$ the trajectory of the agent $A_{j}$ starting from its initial cell $c_{j}\left(0\right)$ and up to the cell $c_{j}\left(t\right)$ occupied at time t. Located at cell $c_{j}\left(t\right)$ the agent makes a decision regarding its next location, following a certain policy $\pi_{j}\left(P\right)$ that prescribes how to choose the next cell $c_{j}\left(t+1\right)$ given a probability map P. For simplicity and tractability, we assume that policy $\pi_{j}\left(P\right)$ for each agent $A_{j}$ does not depend on the time and for any t is specified by the applied probability map P that holds the aggregated information on the location of the targets as a function of past movements of the agents. The result of the application of the policy $\pi_{j}\left(P\right)$ is an action ${𝕒}_{j}\left(t\right)$ that controls the agent movement from the current cell $c_{j}\left(t\right)$ to the next cell $c_{j}\left(t+1\right)$. More precisely, the policy is a function $\pi_{j}:P\to 𝕒\left(A_{j}\right)$, where $𝕒\left(A_{j}\right)$ is a set of possible actions of the agent $A_{j}$, and an action is defined by a function ${𝕒}_{j}\left(t\right):C\to C$ that specifies the choice of the agent’s positions. Assuming that the actions provide an unambiguous choice of the agent’s cells, the required solution is to define the function $\pi_{j}$.

Assume that there are ξ targets, ξ<n, distributed somewhat over the domain, and recall that the probability $p_{s_{i}=1}^{global}\left(t\right)$ defined by Equation (6) is the probability of detecting the targets in cells $c_{i}$, i=1, 2, …,n, by the group of the agents. Both the number of the targets ξ and the global probability $p_{s_{i}=1}^{global}\left(t\right)$ are unknown to the agents; in real situations, the former value either cannot be obtained, or its knowledge requires additional efforts, while the specification of the latter value requires a central unit that obtains data from all the agents, a requirement which can be practically challenging in many applications. However, we use these values as parameters for simulations and sensitivity analysis to demonstrate that better results can be provided by a separate usage of the agents’ probability maps, such that the use of a central unit is often unnecessary.

Denote by $T_{\theta }\left(p|\pi_{1}\left(P\right),\pi_{2}\left(P\right),\ldots ,\pi_{m}\left(P\right)\right)$ the time required to detect the target θ, θ=1,2,…,ξ, with probability p given the agents’ policies $\pi_{1}\left(P\right),\pi_{2}\left(P\right),\ldots ,\pi_{m}\left(P\right)$. Then, the goal is to define such policies that result in minimal time for detecting the last target, that is

(11) $$\left(\pi_{1}^{*}\left(P\right),\pi_{2}^{*}\left(P\right),\ldots ,\pi_{m}^{*}\left(P\right)\right)=\underset{\left(\pi_{1}\left(P\right),\pi_{2}\left(P\right),\ldots ,\pi_{m}\left(P\right)\right)}{\mathrm{argmin}}\underset{\theta =1,2,\ldots ,\xi }{\max}T_{\theta }\left(p\right).$$

Note that this is an NP-hard problem that can’t be solved directly by conventional linear or integer mathematical programming [4,6]. In order to approximate the policies $\pi_{1}^{*}\left(P\right),\pi_{2}^{*}\left(P\right),\ldots ,\pi_{m}^{*}\left(P\right)$ in a tractable manner, we evaluate three different approaches:

- Maximizing of the expected information gain (EIG) locally - over the cells that are reachable to each of the agents in a single move;
- Heading the agents toward the center of view (COV), that is the point that provides maximum expected information-gain over all the cells in the domain;
- Heading the agents toward the center of distribution, also known as the center of gravity (COG), that is defined by the first moment of the probability map.

Notice that the last approach is a greedy heuristic that requires minimal computation efforts, while the first two approaches are more complicated heuristics that require the computation of the possibilities in the local or the global neighborhoods of each agent.

For the aims of comparisons, we also consider a case where static agents remain in their initial places and an agent that accumulates signals received from the targets while being governed by the brute force learning rule. We apply it for a single agent only, since this case is extremely demanding computationally.

The expected information gain EIG(j,k,t) for each sensor ${𝕤}_{jk}$ of the agent $A_{j}$ at time t is defined by the sum of the Kullback–Leibler (KL)divergence measures between the sensor probability map $P^{sensor}\left(j,k,t|{𝕒}_{j}\right)$ as obtained after executing a chosen action ${𝕒}_{j}$ by the agent $A_{j}$ and the sensor probability map $P^{sensor}\left(j,k,t|O\right)$ obtained without execution of any action; where such a null action is denoted by O. Then, the expected information gain, EIG(j,k,t), is defined as

(12) $$EIG\left(j,k,t\right)=D_{KL}\left(P^{sensor}\left(j,k,t|{𝕒}_{j}\right)||P^{sensor}\left(j,k,t|O\right)\right),$$

where $D_{KL}\left(p||q\right)=\sum_{x}p\left(x\right)\log\left(p\left(x\right)/q\left(x\right)\right)$ and logarithm is taken to the base of 2; thus, the distance $D_{KL}$ is represented by the average number of bits. Certainly, instead of the KL distance that is a pseudo-metric in the probability distributions space, other information-theoretic metrics can be used. In particular, EIG can be defined by the Jensen–Shannon divergence as the average of the KL distances $\frac{1}{2}D_{KL}\left(p||M\right)+\frac{1}{2}D_{KL}\left(q||M\right),\;M=\frac{1}{2}\left(p+q\right)$, or by the use of Ornstein or Rokhlin metrics (for application of such metrics to search problems see [4]). Nevertheless, here we use the conventional definition of the EIG, and since the heuristics do not need the metric properties of the probability distributions space, we do not require the distance function to be a formal metric.

At the agent’s level, the expected information gain EIG(j,t) is defined by the sum of the expected information gains for each sensor ${𝕤}_{jk}$, that is

(13) $$EIG\left(j,t\right)=\sum_{k=1}^{l}EIG\left(j,k,t\right).$$

Similarly, for the group of m agents, the expected information gain EIG(t) at time t is

(14) $$EIG\left(t\right)=\sum_{j=1}^{m}EIG\left(j,t\right).$$

Notice that instead of calculating the KL distances for each agent based on his own map as well as calculating the global probability maps over all agents (Equations (5) and (6), respectively), the expected information gains of higher levels are calculated by the sums of expected information gains of the lower levels. Thus, the EIG of the group is calculated as a sum of the EIGs of the agents, and the EIG of the agent is calculated as a sum of the EIGs of its sensors. Such a definition follows a line of additive property of information and leads to essentially simpler computations.

Using the EIGs, the agent’s decision-making follows the maximization of the EIG measure, that is

(15) $${𝕒}^{*}\left(t\right)=\underset{{𝕒}_{j}}{\mathrm{argmax}}EIG\left(t\right).$$

For example, while located in any cell $c_{i},$ an agent can choose one of nine movement possibilities: make a step forward, backward, left, right, left-forward, left-backward, right-forward, right-backward, or stay in its current cell. Then, by Equation (15) the agent chooses such a movement that results in obtaining the maximum expected information gain about the targets’ locations.

The sensor probabilities $p_{s_{i}=1}^{sensor}\left(j,k,t|{𝕒}_{j}\right)$ given the agent action ${𝕒}_{j}$ can be defined by using either the global probability map, $P^{global}\left(t\right),$ or by using the agent probability map, $P^{agent}\left(t\right)$. In the former case, the sensor probabilities are:

(16) $$p_{s_{i}=1}^{sensor}\left(j,k,t|{𝕒}_{j}\right)=p_{s_{i}=1}^{global}\left(t\right)\times Pr\left\{\tilde{a}\left(c_{i},t\right)=1│s_{i}\left(t\right)=1\right\}\times \exp\left[-d\left(c_{i},c\left({𝕒}_{j}\right)\right)/\lambda_{jk}\right],$$

(17) $$p_{s_{i}=1}^{sensor}\left(j,k,t|O\right)=p_{s_{i}=1}^{global}\left(t\right)\times Pr\left\{\tilde{a}\left(c_{i},t\right)=1│s_{i}\left(t\right)=1\right\}\times \exp\left[-d\left(c_{i},c\left(O\right)\right)/\lambda_{jk}\right],$$

while in the latter case these probabilities are defined as follows

(18) $$p_{s_{i}=1}^{sensor}\left(j,k,t|{𝕒}_{j}\right)=p_{s_{i}=1}^{agent}\left(j,t\right)\times Pr\left\{\tilde{a}\left(c_{i},t\right)=1│s_{i}\left(t\right)=1\right\}\times \exp\left[-d\left(c_{i},c\left({𝕒}_{j}\right)\right)/\lambda_{jk}\right],$$

(19) $$p_{s_{i}=1}^{sensor}\left(j,k,t|O\right)=p_{s_{i}=1}^{agent}\left(j,t\right)\times Pr\left\{\tilde{a}\left(c_{i},t\right)=1│s_{i}\left(t\right)=1\right\}\times \exp\left[-d\left(c_{i},c\left(O\right)\right)/\lambda_{jk}\right],$$

where $c\left({𝕒}_{j}\right)$ is the agent’s location after conducting the action ${𝕒}_{j}$ and c(O) is the agent’s location if it decides to avoid conducting any action, i.e., staying in its current location.

A second approach to govern the agent’s action implements the center of view (COV) measure, aiming at the grid point that provides maximum expected information gain over all the cells in the domain. In other words, the difference between information, which the agent obtains in its current position, versus the information, which it expects to obtain while being located at the COV point, reaches its maximum. Formally, it means that in contrast to EIG that is calculated over neighboring locations (thus, eight points around the current agent’s location and its current point), the COV is based on a calculated EIG over all the points in the domain. Thus, instead of calculating the sensor probabilities by Equations (16)–(19) using distances $d\left(c_{i},c\left({𝕒}_{j}\right)\right)$ and, for the COV calculation, the sensor probabilities are defined by using the distances $d\left(c_{i},c_{\eta }\right)$ between the cell $c_{i}$ and other points in the domain, $c_{\eta }$, η=1,2,…,n, that can be considered as candidate locations of the COV. In parallel to Equations (16) and (18) in this case, we have

(20) $$p_{s_{i}=1,G}^{sensor}\left(j,k,t|c_{\eta }\right)=p_{s_{i}=1}^{global}\left(t\right)\times Pr\left\{\tilde{a}\left(c_{i},t\right)=1│s_{i}\left(t\right)=1\right\}\times \exp\left[-d\left(c_{i},c_{\eta }\right)/\lambda_{jk}\right],$$

(21) $$p_{s_{i}=1,A}^{sensor}\left(j,k,t|c_{\eta }\right)=p_{s_{i}=1}^{agent}\left(j,t\right)\times Pr\left\{\tilde{a}\left(c_{i},t\right)=1│s_{i}\left(t\right)=1\right\}\times \exp\left[-d\left(c_{i},c_{\eta }\right)/\lambda_{jk}\right].$$

If the agent chooses to stay in its current location, then the distance is defined as above by $d\left(c_{i},c\left(O\right)\right)$ and the sensor probabilities are calculated by Equations (17) and (19).

By the use of these sensor probabilities, the $EIG_{\eta }$ is defined in parallel to the EIG:

(22) $$EIG_{\eta }\left(j,k,t\right)=D_{KL}\left(P^{sensor}\left(j,k,t|c_{\eta }\right)||P^{sensor}\left(j,k,t|O\right)\right),$$

(23) $$EIG_{\eta }\left(j,t\right)=\sum_{k=1}^{l}EIG_{\eta }\left(j,k,t\right),$$

(24) $$EIG_{\eta }\left(t\right)=\sum_{j=1}^{m}EIG_{\eta }\left(j,t\right),$$

and the COV is the point, in which $EIG_{\eta }$ reaches its maximum, that is

(25) $$COV\left(t\right)=\underset{c_{\eta }}{\mathrm{argmax}}EIG_{\eta }\left(t\right).$$

Finally, the center of gravity (COG), which is the first moment of the probability map, the following calculations are used. In a two-dimensional domain, the location of each cell $c_{i}$, i=1, 2, …,n, is defined by two coordinates, $c_{i}=\left(x_{i},y_{i}\right)$. In addition, recall that $s_{i}=s\left(c_{i}\right)\in \left\{0,1\right\}$ stands for the state of the cell $c_{i}$. Then, the coordinates of the COG for the axes are

(26) $$COG_{x}\left(t\right)=\sum_{i=1}^{n}x_{i}\times p_{s_{i}=1}^{global}\left(t\right)/\sum_{i=1}^{n}p_{s_{i}=1}^{global}\left(t\right),.$$

(27) $$COG_{y}\left(t\right)=\sum_{i=1}^{n}y_{i}\times p_{s_{i}=1}^{global}\left(t\right)/\sum_{i=1}^{n}p_{s_{i}=1}^{global}\left(t\right),$$

and the final location of the COG is obtained by rounding the values $COG_{x}\left(t\right)$ and $COG_{y}\left(t\right)$ to the closest integers that is

(28) $$COG\left(t\right)=\left(\left[COG_{x}\left(t\right)\right],\left[COG_{y}\left(t\right)\right]\right).$$

Notice that since we consider only the states $s_{i}=1$, the sum of the probabilities in the denominator differs from the unit and varies with time and with the number of targets.

Since both here and in the previous case the desired points COG(t) and COV(t) can be located far from the current agent’s location, the agent follows toward these points by steps and changes its direction with respect to the changes of the coordinates of both COG(t) and COV(t).

## 5. Policies Control and Brute Force Learning

In order to control the obtained policies, we apply the simple look-backward method. Based on this method, the control of the agents’ policies is conducted as follows.

Let the global probability map at time t−1 be $P^{global}\left(t-1\right)$ and assume that, following the chosen policies $\pi_{j}\left(P^{global}\left(t-1\right)\right)$, j=1,2,…, m, the agents made a decision and conducted corresponding actions. Then, at time t, each of the agents is located in a new cell and following the observations from these cells the global probability map $P^{global}\left(t\right)\;\mathrm{is}\;\mathrm{constructed}$. The value

(29) $$V_{\pi }\left(t\right)=D_{KL}\left(P^{global}\left(t\right)||P^{global}\left(t-1\right)\right),$$

is the actual information gain that was obtained by the actions defined by the policy $\pi =\left(\pi_{1},\pi_{2},\ldots ,\pi_{m}\right)$.

**Figure 2:**
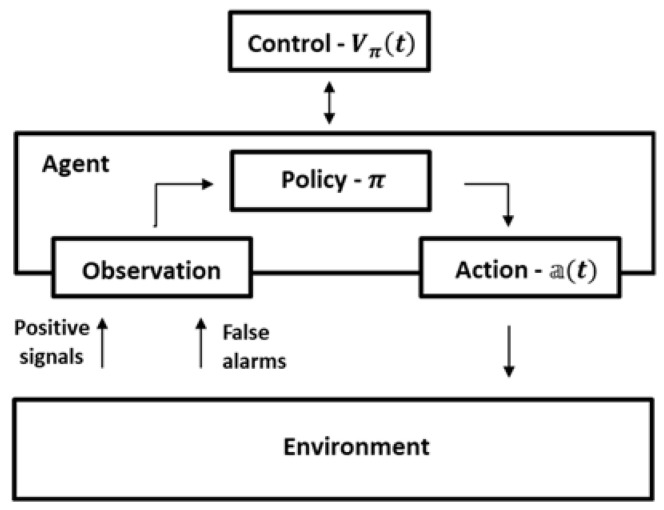
Schema of the agent’s decision-making and control.

The defined decision-making process and control method are illustrated in Figure 2.

Based on the policy π, at time t each agent makes a decision regarding its action. After the action and the corresponding movement, the agent observes the environment, and following the obtained sensor maps, the agent map and the global map are refined. In parallel, in order to control the agent’s policy, a value of information gain $V_{\pi }\left(t\right)$ is calculated.

The calculated information gain $V_{\pi }\left(t\right)$ indicates the efficiency of the applied policy, and it is used as a comparative measure of the agents’ decisions: its accumulated value up to some time T gets larger as the agents’ policies are more efficient in the sense of the quantity of the obtained information about the targets’ locations. Accordingly, the best policy can be defined as follows:

(30) $$\pi^{*}\left(T\right)=\underset{\pi }{\mathrm{argmax}}\sum_{t=1}^{T}V_{\pi }\left(t\right),$$

where $\pi^{*}\left(T\right)$ denotes the best policy among all the combinations of the agents’ policies, $\pi_{j}$, j=1,2,…, m, that can be applied to the available global maps till time T.

The learning measure is also based on the actual information gain, but in this case, it is defined over the actions $𝕒=\left({𝕒}_{1},{𝕒}_{2},\ldots ,{𝕒}_{m}\right)$ that were chosen by the agents, namely:

(31) $$V_{𝕒}\left(t\right)=D_{KL}\left(P^{global}\left(t|𝕒\right)||P^{global}\left(t|O\right)\right),$$

where $P^{global}\left(t|𝕒\right)$ stands for the global probability map obtained after performing the actions of all the agents, and $P^{global}\left(t|O\right)$ is the global probability map, if all the agents stay at their current locations.

The selection of actions is conducted as follows. Assume that at time t the agents are at their locations and observe a certain global probability map $P^{global}\left(t\right)$. Then, for each combination $𝕒=\left({𝕒}_{1},{𝕒}_{2},\ldots ,{𝕒}_{m}\right)$ of their actions and for the null action $O=\left(O_{1},O_{2},\ldots ,O_{m}\right)$, global maps $P^{global}\left(t|𝕒\right)$ and $P^{global}\left(t|O\right)$ are obtained and the average information gain $V_{𝕒}\left(t\right)$ for the combination 𝕒 is specified. The best combination of actions is defined by the maximal value $V_{𝕒}\left(t\right)$, that is

(32) $${𝕒}^{*}\left(t\right)=\underset{𝕒}{\mathrm{argmax}}V_{𝕒}\left(t\right).$$

It is clear that this is the brute force learning that requires a consideration of all possible combinations of actions for all the agents with a large number of iterations. Thus, in practice, such a policy cannot be performed for a large number of agents and actions. However, for a single agent, this learning step can be performed in a relatively short time by available computation resources.

In the considered work, the brute force learning is used as a reference for the evaluation of the suggested methods.

## 6. Numerical Simulations and Analysis

Numerical simulations were implemented using the Python programming language, executed by a regular PC Intel I5 8265U processor. In all the cases, unless defined specifically, the run times of the algorithms are indicated by the numbers of iterations. Also, in all the tables, the policies are measured with respect to the defined approaches: expected information gain (EIG), center of view (COV), and center of gravity (COG), as presented in Section 4.

In the simulations, the search is conducted over a gridded square domain of size $n=n_{x}\times n_{y}$ cells, and it is assumed that each agent and each target can occupy only one cell in the domain. In the simulations, different setups included different numbers m of the agents and different numbers l of the targets. Also, we assume that there are two types of sensors while each agent $A_{j}$ is equipped with two sensors ${𝕤}_{j1}$ and ${𝕤}_{j2}$ of different types with corresponding sensitivities $\lambda_{j1}$ and $\lambda_{j2}$. Both true alarms and false alarms are sent with respect to the sensors’ types and are perceived separately by each of the two sensor types.

Following the goal of finding such policies that result in a minimal time of detecting the last target (see Equation (11)), we determined the maximal simulation time by

(33) $$T_{\max}\left(p\right)=\underset{\theta =1,2,\ldots ,\xi }{\max}T_{\theta }\left(p\right),$$

where, as indicated above, $T_{\theta }\left(p\right)$ is the time required to detect the target θ, θ=1,2,…,ξ, with probability p. Then, the policies that result in a minimum time $T_{max}\left(p\right)$ given probability p are the best policies. In the simulations, we used the probability p=0.95.

To reduce systematic errors, the results presented below were obtained by averaging the outcomes of five repeated trials, each of which contained thirty sessions, executed with the same parameters, with the same initial locations of both the searchers and the targets. The true and false alarms in the sessions were generated by the same uniform distribution with a random seed for each trial.

### 6.1. Detection by a Single Agent

Let us start with a small illustrative example of detection by a single agent $A_{1}$. In order to simulate the brute force learning defined by the Equations (31) and (32), we considered a small domain of the size n=20×20=400 cells. A broadcast of the false alarms was distributed uniformly over the domain, and the frequency of sending false alarms from all 400 cells was 100 false alarms per second for each type of sensor, that is, on average 1/4 alarms per second from each cell to each type of sensor. The sensitivities of the sensors are $\lambda_{11}=\lambda_{12}=10$.

In the first setting, the single agent $A_{1}$ was detecting l=3 targets located in the cells with coordinates $c_{1}=\left(11,16\right)$, $c_{2}=\left(0,14\right)$ and $c_{3}=\left(7,1\right)$; the starting position of the agent was c(0)=(20,8). The results of the simulation trials are summarized in Table 1.

As expected, the best result, which leads to the minimum of $T_{max}\left(0.95\right)$ is obtained by the brute force learning, while the times obtained by the policies based on the expected information gain (EIG), the center of view (COV), and the center of gravity (COG) are close to this best result. Notice that since the detection is conducted by a single agent, the results obtained by the COV and COG policies are equal.

**Figure 3:**
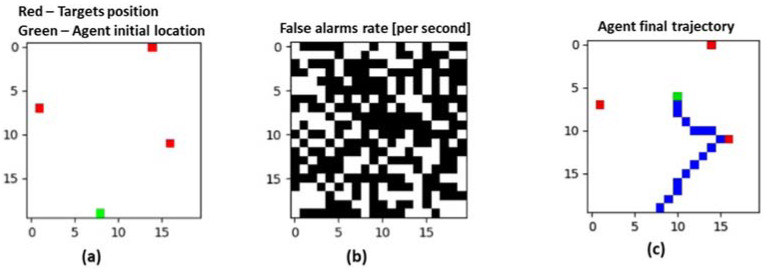
Activity of a single agent detecting three targets setting. (**a**)—The targets’ positions (red squares) and the agent’s initial location (green squire). (**b**)—100 false alarms per second for each type of sensor (white color indicates false alarm). (**c**)—The agent’s trajectory and final position.

Figure 3 illustrates the activity of a single agent detecting three targets using the center of view (COV) policy.

The values of the accumulated information gain (see Equation (30)

(34) $$V_{\pi }\left(T\right)=\sum_{t=1}^{T}V_{\pi }\left(t\right),$$

that characterizes the effectiveness of the policy for the simulated policies are presented in Table 2. The time T=17 is the minimal time of detecting the last target based on the brute force policy.

With respect to the detection time, the maximal accumulated information gain $V_{\pi }\left(T\right)$ is obtained by the brute force learning policy, while the EIG, COV, and COG policies result in the accumulated information gains that are close to the maximum.

In the next simulation, a single agent $A_{1}$ is detecting l=5 targets located in the cells of the following coordinates: $c_{1}=\left(11,16\right)$, $c_{2}=\left(0,14\right)$, $c_{3}=\left(7,1\right)$, $c_{4}=\left(5,3\right)$ and $c_{5}=\left(11,15\right)$; the starting position of the agent is c(0)=(19,8). The results of the simulation trials are summarized in Table 3.

Similar to the previous case, the best result is obtained by the brute force learning, while the times obtained by the policies based on the center of view (COV) and the center of gravity (COG) are close to this best result. However, for this case with larger number of targets, one can already notice that the policy based on the expected information gain (EIG) is worse than the first three policies.

The relations between the values of the accumulated information gain, in this case, are again similar to the case of the detection of three targets. The best result is provided by the brute force learning policy. Then, the results of the COV and the COG policies obtain close to the brute force, while the EIG policy is less effective. The worst results are obtained by the static agent.

### 6.2. Detection by Multiple Agents

Now let us consider detection by a group of agents that can share information and consequently use probability maps of each other as well as a global probability map, which represents the knowledge of the group.

Since in this case, we have not considered the brute force learning, in the simulations, we used a greater domain of size n=40×40=1600 cells. As mentioned above, a broadcast of the false alarms was distributed uniformly over the domain. However, the frequency of sending false alarms from all 1600 cells was 400 false alarms per second for each type of sensor that is 1/4 alarms per second on average from each cell to each type of sensor. It is assumed that each agent $A_{j}$ is equipped by the sensors of two types. The sensitivities of the sensors of each type are denoted by $\lambda_{j1}$ and $\lambda_{j2}$.

Recall that located in a cell, each agent can choose one of nine alternatives, while if the agent is located at the border of the domain, then the number of alternatives is smaller due to boundary conditions of the map.

In addition to the considered policies that are based on (i) expected information gain (EIG), (ii) center of view (COV), and (iii) center of gravity (COG), in the following simulations, we also distinguish decision-making under several scenarios:(i) when relying on the agent probability map versus (ii) when relying on the global probability map, as well as (i) selection of actions by each agent separately or (ii) selection of actions mutually by all the agents in the group. The use of the maps and the selection of the actions for the different policies are summarized in Table 4.

The table presents the fact that when applying the EIG policy, from its current cell the agent can either move to one of eight neighboring cells or stay in its current location. When applying the COV policy the agent can choose one of n cells as a desired center of view, and when applying the COG policy the agent can consider only one cell as the COG cell.

For consistency, let us start with the same setting as in the previous simulations, namely by detection of l=5 targets located at the cells with coordinates $c_{1}=\left(4,34\right)$, $c_{2}=\left(6,23\right)$, $c_{3}=\left(37,3\right)$, $c_{4}=\left(32,13\right)$ and $c_{5}=\left(2,5\right)$. These simulations are conducted for m=2 agents, each of which is equipped by two sensors with respective sensitivities $\lambda_{j1}=\lambda_{j2}=10$, j=1,2. Initial positions of the agents are $c_{1}\left(0\right)=\left(25,3\right)$ and $c_{2}\left(0\right)=\left(20,9\right)$. The results of the simulations when using different policies are presented in Table 5 (cf. Table 3).

**Figure 4:**
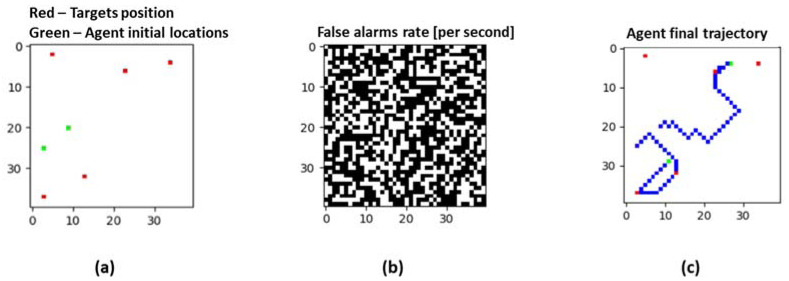
Activity of two agents detecting five targets setting. (**a**)—The targets’ positions (red squares) and the agents’ initial locations (green squares). (**b**)—Map of 400 false alarm signals per second for each type of sensor (white color indicates false alarm). (**c**)—The agent trajectories and final positions.

Figure 4 illustrates the activity of two agents detecting five targets when using the expected information gain (EIG) with agent map/agent action policy.

Results of simulations using different policies are presented in Table 5 (cf. Table 3).

As expected, the worst results are obtained by the static agents. The best results are provided by the policies based on the center of view (COV) and center of gravity (COG). As indicated above, these policies result in the same detection times. Finally, the EIG policy, as seen above, results in a longer search than the COV and the COG policies, yet, better than the static agent policy. 

In addition, notice that the decision-making policies that are based on the agent map provide significantly better results than the policies based on the global map. In other words, in the detection tasks, more information is not always better, unless actions between the agents can be synchronized. The reason for this result is the following. Relying on a single global map, all agents aim at the same preferable regions with the higher probabilities of detecting the targets while ignoring the regions with the lower probabilities. However, because of the existence of both false positive and false negative errors, the targets can appear in those ignored regions, to which the agents return only after the unsuccessful detection in the preferable regions. All these movements waste a lot of time. In contrast, while using the agent maps, each agent considers its local region and continues to the other regions only after unsuccessful detection in its close neighborhood. In such a manner, the agents divide the task and conduct the detection process in parallel in different regions. At the same time, the global map is used for terminating the detection for all the agents.

Finally, notice that a better choice of actions is provided by applying the group action. However, since it requires strong computation power without a significant improvement of the detection time, this approach is less attractive for practical tasks.

The accumulated information gain for this simulated detection of l=5 targets by m=2 agents is presented in Table 6; the CPU times required for such detection are presented in Table 7.

The obtained results support the previous observations. The higher accumulated information gain is obtained by the COV and the COG policies, the EIG policy obtains the worse results, and the lowest gain is obtained by the group of static agents. Also, better results are achieved by the decision-making policies based on the agent maps, and the better choice of actions is provided by the use of group action.

In order to represent the relation between the detection efficiency and the sensors’ sensitivity, a similar detection scenario was simulated for the agents that are equipped by sensors with different sensitivities $\lambda_{1k}=12$ and $\lambda_{2k}=8$, k=1,2. Table 8 presents the detection times $\;T_{max}\left(0.95\right)$ and that accumulated information gains $V_{\pi }\left(100\right)$ for this scenario.

A comparison of the obtained times and information gains with the results presented in Table 6 and Table 8, respectively, show that in general, the change of the sensors’ sensitivities preserves the already considered trends in the efficiencies of the policies. At the same time, it stresses an advantage of the group action relative to the actions by each agent separately.

**Figure 5:**
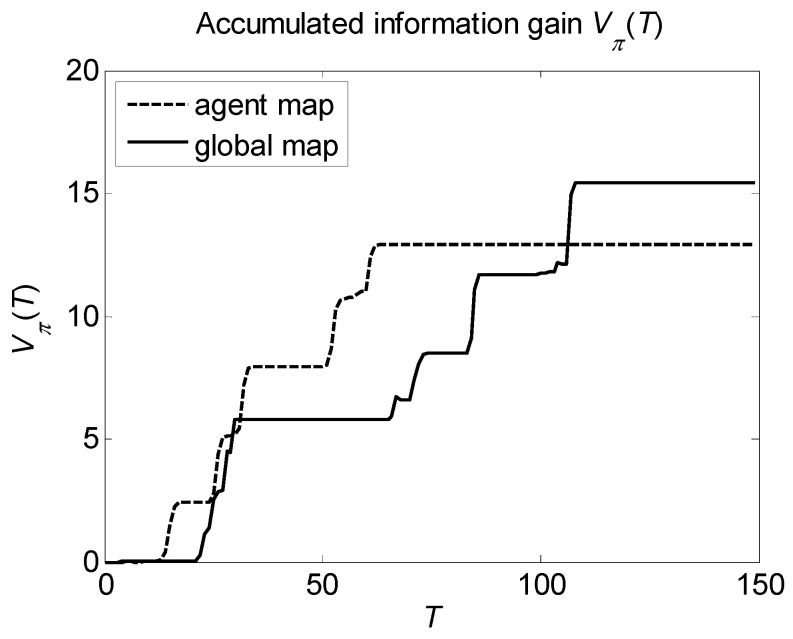
Dependence of the accumulated information gain $V_{\pi }\left(T\right)$ on the detection time T for the COV policy with agent action choice; l=5 targets, m=2 agents, and sensors’ sensitivities $\lambda_{1k}=12$ and $\lambda_{2k}=8$, k=1,2.

Finally, let us consider the dependence of the accumulated information gain on the detection time. An example of such functions is shown in Figure 5, where we used the results of the previous simulation of detecting l=5 targets by m=2 agents with different sensors sensitivity $\lambda_{1k}=12$ and $\lambda_{2k}=8$, k=1,2, following the COV policy with agent action choice.

It is seen that in the beginning of the search process, the policy based on the agent map accumulates information faster than the policy based on the global map. However, as the search process continues, the accumulated information gain obtained by the global map policy converges to a value which is significantly greater than the one to which the agent map policy converges.

For validation of the presented results, further simulations were conducted for different settings. The obtained detection times and information gains demonstrate the same trends of the policies’ efficiency. In addition, it was found that as the number of the agents gets larger, the difference between the best COV policy and the nearly best COG and EIG policies is increasing.

## 7. Discussion

The paper presents three heuristic techniques for navigation of autonomous agents searching for hidden targets in the presence of false positive and false negative errors. Two of these heuristics are based on the expected information gain calculated over the local neighborhood of each agent (EIG policy) or relative to the center of view (COV policy), and the third heuristic uses the center of gravity (COG policy) of the targets’ location probabilities. In order to make decisions regarding the next movements, the agents use either their own probability maps or a global probability map.

The simulations show that in short-term detection processes, the policies based on the agent map outperform the policies based on the global map, both while the agents’ movements are not centrally synchronized (individual decision-making) and while the agents’ actions are definitely synchronized (collective decision-making in the group). However, in the long-term detections, the accumulated information gain during the search process when using the global map policy was significantly larger than the accumulated information gain during the search process when using the agents’ maps policy. Probably, the reason for such a result is the following: while using the global map, the agents calculate the information gain also taking into account the not relevant information based on the false alarms, and while using the agents’ maps the influence of such alarms is lower and, consequently, the accumulated information gain is lower as well.

Detection using the EIG policy demonstrated lower efficiency (in terms of detection time) than the COV and COG policies while using both the agents’ and the global probability maps. The main reason for such a result is the following. On the one hand, the EIG policy does not always recognize what should be the next step since the differences in the expected information gain obtained by staying in the current cell and by moving to a neighboring cell are extremely small and cannot be used for a reasonable selection of the actions. On the other hand, in order to reveal the center of view, the COV policy requires the agent to check all the cells in the domain and, consequently, succeeds to find a significant change in the expected information gain.

While using the sensors with equal sensitivities, the COV and COG policies result in a close or even equal detection times. Therefore, since the COG policy has an extremely lower computational complexity than the COV policy, it should be preferable when the agents are equipped with similar sensors. However, if the sensitivities of the sensors are different, then the COV policy is significantly better.

As expected, a decision-making heuristic which relies on group actions leads to better performance than the one which relies on single agents’ actions, yet, the first heuristic requires much greater computation efforts. In order to shorten the running time, the number of calculations can be decreased by using some probability threshold. Thus, at each step of the computation, the cells with probabilities lower than the threshold are ignored, thus the number of calculations is reduced without significantly influencing the quality of the search results. 

Finally, notice that the results obtained when using the presented techniques are close to the results obtained by the optimal brute-force learning method. Such a comparison both validates the suggested methods and demonstrates that, for the detection over large domains, where due to intractable computation complexity the brute force learning cannot be used, these methods might provide sub optimal results by reasonable computation efforts in reasonable running time. 

## 8. Conclusions

In the paper, we considered the problem of detection of multiple targets by the group of mobile agents that directly extends the classical Koopman search problem [2]. In contrast to many known algorithms, we addressed detection with both false positive and false negative detection errors.

The suggested solution implements three different levels of the agent’s knowledge about the targets’ locations: information that is available to the group of agents, information available to a single agent, and information obtained by a single on-board sensor of an agent.

For these settings, we considered three decision-making policies based on different considerations of the expected information gain, which can be obtained by the agent in its next step. Namely, the policies considered a local neighborhood of the agent, a location of the “center of view” from which the agents can obtain maximum information using their sensors, and a location of the “center of gravity” of the targets’ probability map.

The results obtained using the suggested policies were compared against the results provided by the worst policy, in which the agents are static, as well as the best policy of the brute force learning when tractable.

Simulations of the suggested solutions demonstrate that the best results among the constructed policies are obtained by a policy which is based on the center of view. Close results are provided by the policy based on the use of the center of gravity, and the worse results, yet sometimes satisfactory, were achieved by the policy based on the expected information gain over a local neighborhood of the agent.

In addition, it was found that in the considered problem including both false positive and false negative detection errors, decision-making policies based on the agent maps provide significantly better results than the policies based on the global map.

The best search policy under the considered settings was obtained by relying on group actions, at the expense of having a strong computation complexity, without significantly improving the detection time relative to the suggested heuristics. This observation makes this approach less attractive to implement.

Finally, it was demonstrated that the policies based on the agent map are more effective for the detection in a given short period of time, while in a long-term detection, the policies based on the use of a global map result in better outcomes in terms of accumulated information gain.

The constructed algorithms and software can form a basis for the further development of the proposed methods as well as other methods related to probabilistic search and detection. These methods can be used directly for practical applications in various fields, such as smart cities, military applications, and autonomous vehicles.

## Figures and Tables

**Table 1:** Times required for the detection of the last among l=3 targets with probability p=0.95 by a single agent implementing different policies.

Detection Policy	Detection Times			
	First Target	Second Target	Third Target	$T_{max}\;\left(0.95\right)$
Static agen	15	72	15	72
EIG	15	19	11	19
COV	8	18	15	18
COG	8	18	15	18
Brute force learning	10	17	14	17

**Table 2:** Accumulated information gain of the detection of l=3 targets by a single agent for the times T=10 and T=17.

Detection Policy	$\mathrm{Accumulated}\;\mathrm{Information}\;\mathrm{Gain}\;V_{\pi }\left(T\right)$	
	T=10	T=17
Static agent	3.6	4.1
EIG	4.2	6.1
COV	4.4	6.4
COG	4.4	6.4
Brute force learning	4.6	6.8

**Table 3:** The times required for detection of the last among l=5 targets with the probability p=0.95 by a single agent implementing different policies.

Detection Policy	Detection Times					
	First Target	Second Target	Third Target	Fourth Target	Fifths Target	$T_{max}\left(0.95\right)$
Static agent	14	94	29	12	9	94
EIG	9	32	20	13	7	32
COV	14	28	22	10	8	28
COG	14	28	21	10	8	28
Brute force learning	14	25	17	18	11	25

**Table 4:** Decision-making and action choice in the group of m agents act in a domain with n cells.

Decision Making/Action Choice	Number of Alternatives		
	EIG	COV	COG
Agent map/agent action	9m	mn	m
Global map/agent action	9m	mn	1
Global map/group action	$9^{m}$	$n^{m}$	1

**Table 5:** The times required for the detection of the last among l=5 targets with the probability p=0.95 by m=2 agents implementing different policies.

Detection Policy		Detection Times					
		First Target	Second Target	Third Target	Fourth Target	Fifths Target	$T_{max}\left(0.95\right)$
Static agents		400	90	47	27	70	400
EIG	Agent map/agent action	95	37	51	18	60	95
	Global map/agent action	143	123	53	28	109	143
	Global map/group action	138	116	35	28	103	138
COV	Agent map/agent action	88	33	43	18	59	88
	Global map/agent action	126	85	35	28	81	126
	Global map/group action	108	85	37	32	61	108
COG	Agent map	87	33	43	18	59	87
	Global map	126	89	35	28	81	126

**Table 6:** Accumulated information gain in the detection of l=5 targets by m=2 agents for the times T=75 and T=100.

Detection Policy		$\mathrm{Accumulated}\;\mathrm{Information}\;\mathrm{Gain}\;V_{\pi }\left(T\right)$	
		T=75	T=100
Static agents		4.4	6.5
EIG	Agent map/agent action	9.9	13.6
	Global map/agent action	5.7	7.9
	Global map/group action	5.8	8.7
COV	Agent map/agent action	10.8	14.2
	Global map/agent action	8.4	11.7
	Global map/group action	9.2	12.6
COG	Agent map	10.9	14.2
	Global map	8.4	11.7

**Table 7:** CPU time (sec.) for target detection with probability p=0.95 of l=5 targets by m=2 agents.

Detection Policy		CPU Time (sec)
Static agents		240
EIG	Agent map/agent action	75
	Global map/agent action	180
	Global map/group action	850
COV	Agent map/agent action	220
	Global map/agent action	350
	Global map/group action	3700
COG	Agent map	25
	Global map	32

**Table 8:** Detection times and accumulated information gain in the detection of l=5 targets by m=2 agents with different sensor sensitivities $\lambda_{1k}=12$ and $\lambda_{2k}=8$, k=1,2.

Detection Policy		$T_{max}\left(p=0.95\right)$	$V_{\pi }\left(T=100\right)$
Static agents		300	5.1
EIG	Agent map/agent action	81	13.6
	Global map/agent action	144	6.2
	Global map/group action	129	9.0
COV	Agent map/agent action	63	12.9
	Global map/agent action	109	11.7
	Global map/group action	96	12.4
COG	Agent map	67	12.9
	Global map	109	11.9
